# Safety and efficacy of drug-eluting beads versus conventional bronchial arterial chemoembolization for lung cancer: a systematic review and meta-analysis

**DOI:** 10.3389/fonc.2026.1881323

**Published:** 2026-07-21

**Authors:** Zezhong Gong, Yali Liu, Yusheng Song, Xinghai Li, Yunfei Tian, Jing Li, Chaoyi Qi

**Affiliations:** 1 Department of Interventional Radiology and Vascular Surgery, Ganzhou Key Laboratory of Interventional Medicine, Ganzhou Hospital-Nanfang Hospital, Southern Medical University (Ganzhou People’s Hospital), Ganzhou, Jiangxi, China; 2 General Practice Department, Ganzhou Hospital-Nanfang Hospital, Southern Medical University (Ganzhou People’s Hospital), Ganzhou, Jiangxi, China; 3 Taian Hospital of Traditional Chinese Medicine, Shandong University of Traditional Chinese Medicine Affiliated Hospital, Taian, Shandong, China

**Keywords:** bronchial arterial chemoembolization (BACE), drug-eluting bead bronchial arterial chemoembolization, drug-eluting beads, lung cancer, meta-analysis

## Abstract

**Background:**

Conventional bronchial arterial chemoembolization (cBACE), operationally defined in this review as bronchial arterial infusion chemotherapy with or without embolization using non-drug-eluting materials, is used as a locoregional palliative option for selected patients with unresectable lung cancer. Drug-eluting beads bronchial arterial chemoembolization (DEB-BACE) may enhance therapeutic outcomes compared to cBACE, but comparative evidence remains limited.

**Objectives:**

To compare the efficacy and safety of DEB-BACE with cBACE in patients with lung cancer.

**Methods:**

A systematic search was conducted in PubMed, Embase, the Cochrane Library, and Web of Science from their inception to July 15, 2025 for studies comparing DEB-BACE with cBACE. Meta-analysis was performed using Review Manager 5.4 and STATA 16.

**Results:**

Five China-based comparative studies involving 221 patients were included. DEB-BACE was associated with higher pooled ORR at 1–3 months (OR = 5.38, 95% CI: 2.30–12.60) and 6 months (OR = 4.18, 95% CI: 1.90–9.19), as well as higher pooled DCR at 1–3 months (OR = 7.00, 95% CI: 1.99–24.60) and 6 months (OR = 5.40, 95% CI: 2.14–13.62). No statistically significant between-group differences were observed for hemoptysis, fever, chest discomfort, cough/sputum production, or nausea/vomiting.

**Conclusion:**

Current China-based comparative evidence suggests that DEB-BACE may be associated with higher short-term tumor response than conventional comparator treatment without an obvious increase in the assessed procedure-related adverse events. Given the small, mainly observational evidence base and heterogeneous comparator definitions, these findings remain preliminary and warrant confirmation in well-designed multicenter randomized trials.

## 1 Introduction

Lung cancer persists as one of the most prevalent and lethal malignancies worldwide, demonstrating particularly alarming epidemiological patterns in China where it consistently ranks first in both incidence and mortality among all cancers, imposing substantial socioeconomic and healthcare burdens (1). While systemic therapies including platinum-based chemotherapy, tyrosine kinase inhibitors, and immune checkpoint inhibitors have improved outcomes, therapeutic options remain limited for patients with progressive disease after multiple lines of treatment (2, 3).

Bronchial artery embolization (BAE), first described by Remy et al. in 1973 for massive hemoptysis, now achieves contemporary technical success rates of 97.2% (95% CI: 94.1-98.7%) in experienced centers (4). Beyond hemostatic embolization, bronchial arterial infusion chemotherapy and conventional bronchial arterial chemoembolization have been explored as locoregional oncologic approaches for advanced lung malignancies. cBACE delivers chemotherapeutic agents directly to the tumor site via bronchial arteries, potentially increasing local drug exposure while reducing systemic exposure compared with conventional intravenous chemotherapy. This targeted approach minimizes drug exposure to normal tissues, thereby reducing systemic toxicity while maintaining therapeutic efficacy (5).

In recent years, drug-eluting bead bronchial arterial chemoembolization (DEB-BACE) has been increasingly explored in advanced lung cancer treatment. This technique involves intra-arterial delivery of chemotherapy-loaded microspheres into tumor-feeding bronchial arteries, achieving dual therapeutic effects: mechanical occlusion of tumor-feeding vessels and sustained local release of chemotherapeutic agents (6). DEB-BACE has been utilized in the management of advanced lung cancer, particularly in cases complicated by hemoptysis, chemotherapy-refractory disease, or malignant pleural effusion (7, 8). Zhao et al. observed that after initial interventional therapy, patients exhibited effective control of cough, wheezing, hemoptysis, and superior vena cava syndrome, with the incidence of adverse events being less than 20% (9).

Although DEB-BACE has been associated with encouraging tumor response and manageable adverse events in preliminary studies, whether it provides clinically meaningful advantages over conventional comparator treatment remains inadequately defined. In this review, cBACE refers to bronchial arterial infusion chemotherapy with or without embolization using conventional non-drug-eluting materials. Given the additional procedural cost and resource requirements of DEB-BACE, clarifying its incremental benefit over cBACE or conventional comparator treatment is clinically important. This systematic review aims to comprehensively compare the efficacy and safety of DEB-BACE versus cBACE in the treatment of lung cancer.

## 2 Methods

### 2.1 Search strategy

A comprehensive electronic search was performed across multiple databases including PubMed/MEDLINE, Embase, the Cochrane Library, and Web of Science from their respective inception dates through July 15, 2025. The search methodology incorporated both controlled vocabulary (Medical Subject Headings [MeSH] terms) and text word searches using key terms such as: “lung neoplasms,” “lung cancer,” “lung malignancy,” “pulmonary cancer,” “drug-eluting beads,” “DEB-BACE,” “bronchial artery chemoembolization,” “Chemoembolization, Therapeutic,” “chemoembolization,” and “cBACE”. Additionally, we conducted manual searches of bibliographies from identified publications, systematic reviews, and meta-analyses to ensure comprehensive coverage. This systematic review and meta-analysis was designed and reported in accordance with the Preferred Reporting Items for Systematic Reviews and Meta-Analyses (PRISMA) guidelines, and key methodological domains were considered with reference to A Measurement Tool to Assess Systematic Reviews 2 (AMSTAR 2).

### 2.2 Inclusion and exclusion criteria

This systematic review and meta-analysis incorporated randomized controlled trials, prospective cohort studies, and retrospective cohort studies that directly compared DEB-BACE with cBACE in the treatment of lung malignancies. DEB-BACE was defined as bronchial arterial chemoembolization using chemotherapy-loaded microspheres. cBACE was defined as bronchial arterial chemotherapy with or without embolization using conventional non-drug-eluting materials. Embolic materials were recorded only when explicitly described in the source studies; otherwise, they were marked as not reported (NR). English-language articles were eligible if they reported at least one of the following outcomes: (1) Tumor response, evaluated by Objective Response Rate (ORR) and Disease Control Rate (DCR); (2) Treatment-related adverse events (TRAEs). Studies that solely utilize DEB-BACE or compare DEB-BACE with other techniques will be excluded.

### 2.3 Outcomes

The primary endpoints were ORR and DCR. Tumor response was evaluated according to the Response Evaluation Criteria in Solid Tumors (RECIST) version 1.1. Complete response (CR) was defined as the disappearance of all target lung lesions, with a reduction in the short axis of any pathological lymph nodes to below 10 mm. Partial response (PR) was characterized by at least a 30% decrease in the sum of diameters of target lesions, taking as reference the baseline sum of diameters. Progressive disease (PD) referred to at least a 20% increase in the sum of diameters of target lesions compared to the smallest sum recorded during treatment, with a minimum absolute increase of 5 mm, and/or the emergence of one or more new lesions. Stable disease (SD) was defined as a state in which the change in the sum of target lesion diameters did not meet the criteria for either PR or PD. The ORR was calculated as the proportion of patients achieving complete or partial response, expressed as (CR + PR)/(CR + PR + SD + PD) × 100%. Similarly, DCR was defined as the percentage of patients exhibiting complete response, partial response, or stable disease, calculated using the formula (CR + PR + SD)/(CR + PR + SD + PD) × 100%. Hemoptysis, fever, chest discomfort, cough/sputum production, and nausea/vomiting, as part of adverse event, were counted as secondary outcomes.

### 2.4 Data extraction and analysis

Two investigators independently screened the titles and abstracts of retrieved studies. Discrepancies were resolved by a third investigator. Full-text articles of potentially eligible studies were thoroughly reviewed, and those meeting the inclusion criteria were selected for final analysis.

### 2.5 Quality assessment

The Newcastle-Ottawa Scale (NOS) was employed to evaluate the quality of cohort studies. In cases of disagreement between two investigators, a third investigator made the final decision.

### 2.6 Statistical analysis

All statistical analyses were performed using Review Manager 5.4 (Cochrane Collaboration) and STATA 16. Dichotomous outcomes were summarized as odds ratios (ORs) with 95% confidence intervals (CIs), and P < 0.05 was considered statistically significant. Heterogeneity was assessed using the I^2^ statistic. A fixed-effect model was used when heterogeneity was low (I^2^ < 50%); otherwise, a random-effects model was planned. Publication bias was not formally assessed because fewer than 10 studies contributed to each outcome.

## 3 Results

**Figure 1:**
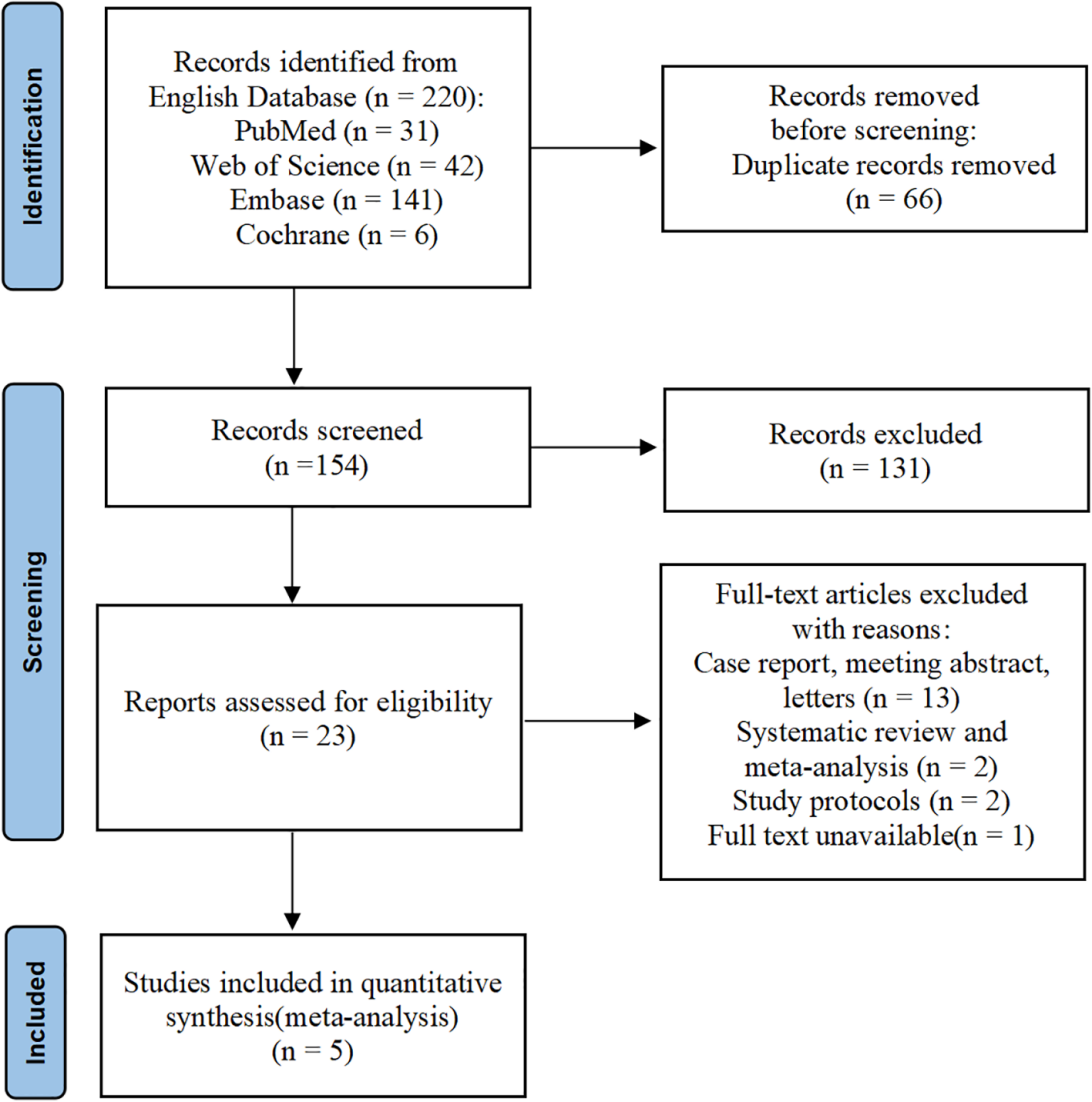
Flow diagram for selection of studies.

Through comprehensive database interrogation, our search strategy initially identified 220 potentially relevant studies exclusively sourced from four major English-language medical databases (PubMed, Embase, Cochrane Library, and Web of Science). Following rigorous screening protocols, 23 full-text articles underwent meticulous evaluation, with five studies ultimately satisfying all predefined inclusion criteria (detailed selection process illustrated in **Figure 1**).

The pooled analysis incorporated data from 221 enrolled participants, including a clinically relevant subset of 94 patients (42.5%) who received DEB-BACE (**Table 1**). The temporal distribution of included publications spanned the contemporary period from 2020 to 2025. Regarding the study design, retrospective cohort studies were predominant, with only one prospective cohort study included. The main tumor types were squamous cell carcinoma and adenocarcinoma, and most patients were at clinical stage III or IV. The drug-eluting microspheres used were mainly of two types: CalliSphere and HepaSphere. The median overall survival (mOS) of patients who received DEB-BACE ranged from 13.7 to 28.5 months, while that of patients who received cBACE ranged from 6.5 to 22.5 months. Reported follow-up durations varied from approximately 8.5 to 16 months.

**Table 1:** General characteristics of the included article in the meta-analysis.

Author	Country	Statistical time	Study design	Diagnosis	Treatment	Quantity	Histological subtype	Neoplasm staging	Embolic material/agent	Chemotherapeutics	MST	mPFS	Follow-up time	AE
Fu et al. (10)	China	2018.01-2020.07	Retrospective cohort study	Lung cancer with hemoptysis	DEB-BACE	14	SCC:11 ADC:2	II:1 III:8	CalliSpheres DEB	Gemcitabine	NR	NR	NR	①③④⑤
							SCLC:1	IV:5						
					cBACE	22	SCC:13 ADC:8	II:2 III:7	NR	Gemcitabine	NR	NR		
							SCLC:1	IV:13						
He et al. (11)	China	2018.10- 2020.01	Retrospective cohort study	Advanced squamous cell lung cancer	DEB-BACE	20	SCC	III:8 IV:12	gemcitabine-loaded DEB	Nedaplatin,	13.7	4.3	8.9	②④⑤
										Gemcitabine				
					cBACE	16	SCC	III:5 IV:11	PVA particles	Nedaplatin,	6.5	3.2		
										Gemcitabine				
Shang et al. (12)	China	2018.08-2019.05	Prospective cohort study	lung cancer	DEB-BACE	30	SCC:22 ADC:7 Others:1	II:3 III:5 IV:22	CalliSpheres DEB	adriamycin	NR	NR	8.5	NR
					Bronchial arterial chemotherapy	30	SCC:20 ADC:9 Others:1	II:5 III:3 IV:22	NR	epirubicin/pirarubicin	NR	NR		
Sun et al. (13)	China	2021.01-2023.04	Retrospectivecohort study	NSCLC	DEB-BACE	18	SCC:9 ADC:9	III:8 IV:10	CalliSphere/HepaSphere DEB	Cisplatin/	19.5	13	16	②③④
										doxorubicin				
					cBACE	30	SCC:11 ADC:19	III:11 IV:19	Gelfoam/gelform particles	SCC: pemetrexed and cisplatin ADC: gemcitabine and cisplatin	12.7	7		
Yu et al. (14)	China	2019.04-2022.12	Retrospectivecohort study	Advanced NSCLC	DEB-BACE	12	SCC:10	III:11	HepaSphere DEB	Cisplatin	28.5	6.95	13.7	①②③⑤
							ADC:2	IV:1						
					cBACE	29	SCC:26	III:23	microspheres	SCC: cisplatin and docetaxel	22.5	3.2		
							ADC:3	IV:6		ADC: gemcitabine and cisplatin				

MST, Median survival time; mPFS, median Progression-Free Survival; AE, Adverse event; SCC, Squamous cell carcinoma; ADC, Adenocarcinoma; SCLC, Small-cell lung cancer; NR, not reported; PVA, polyvinyl alcohol.

AE:①hemoptysis; ②fever; ③chest discomfort; ④cough/sputum production; ⑤nausea/vomiting.

### 3.1 Assessment of included studies

Four cohort studies were rated as high-quality (Newcastle-Ottawa Scale [NOS] score ≥7) (**Table 2**), demonstrating robust methodology in terms of selection, comparability, and outcome assessment. The study by Fu et al. primarily enrolled lung cancer patients complicated with hemoptysis, which may introduce potential selection bias into the reported outcomes. While these studies consistently reported balanced baseline characteristics across study groups, significant variability was observed in the number of surgical interventions performed—a factor that emerged as a critical determinant of patient survival.

**Table 2:** Results of quality assessment using the Newcastle–Ottawa Scale for included cohort studies.

Study	Selection				Comparability	Exposure			Scores
	Adequate definition of cases	Representati-veness of the cases	Selection of controls	Definition of controls	Control for important factors	Ascertainment of exposure	Same method of ascertainment for cases and controls	Non-response rate	Scores
Fu 2022	★	☆	★	★	★☆	★	★	☆	6
He 2023	★	★	★	★	★☆	★	★	☆	7
Shang 2020	★	★	★	★	★☆	★	★	☆	7
Sun 2025	★	★	★	★	★☆	★	★	☆	7
Yu 2023	★	★	★	★	★☆	★	★	☆	7

**★** = 1 point, ☆ = 0 points.

### 3.2 1–3 month ORR and DCR

**Figure 2:**
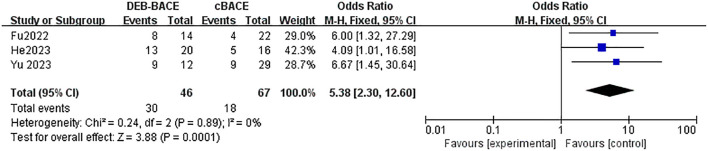
Meta-analysis of ORR between DEB-BACE and cBACE at 1–3 month follow-up.

**Figure 3:**
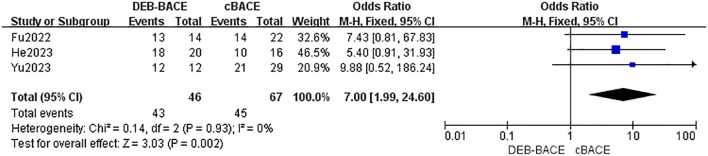
Meta-analysis of DCR between DEB-BACE and cBACE at 1–3 month follow-up.

Three studies involving a total of 113 patients reported short-term follow-up results at 1–3 months, including ORR and DCR. DEB-BACE was associated with a significantly higher ORR than cBACE or conventional comparator treatment (OR = 5.38, 95% CI: 2.30–12.60; P = 0.0001; I^2^ = 0%) (**Figure 2**). A fixed-effects model was employed due to the absence of heterogeneity (I^2^ = 0%). Similarly, DCR at 1–3 months was significantly higher in the DEB-BACE group compared to the cBACE group (OR = 7.00, 95% CI: 1.99-24.60; P < 0.05) (**Figure 3**).

### 3.3 6-month ORR and DCR

**Figure 4:**
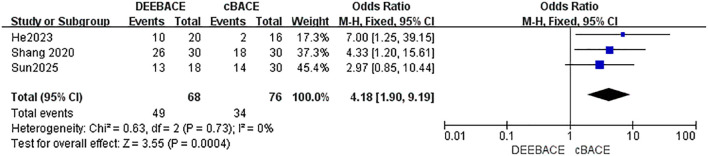
Meta-analysis of ORR between DEB-BACE and cBACE at 6 month follow-up.

**Figure 5:**
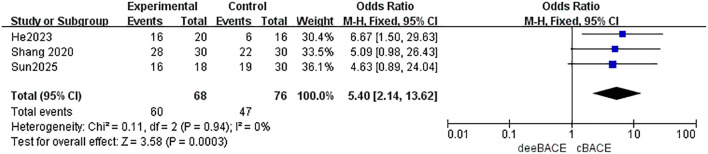
Meta-analysis of DCR between DEB-BACE and cBACE at 6 month follow-up.

Pooled analysis of three studies involving 144 patients showed that DEB-BACE was associated with a higher 6-month ORR than cBACE or conventional comparator treatment (OR = 4.18, 95% CI 1.90 -9.19, P <0.05) (**Figure 4**). Additionally, the DEB-BACE group showed superior DCR at 6 months compared to the cBACE group (OR = 5.40, 95% CI 2.14 -13.62, P <0.05) (**Figure 5**).

### 3.4 Adverse events

**Figure 6:**
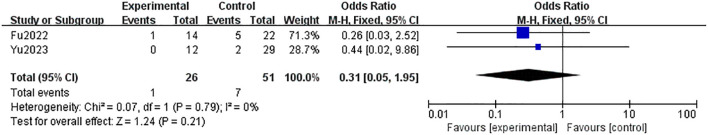
Meta-analysis of hemoptysis between DEB-BACE and cBACE.

**Figure 7:**
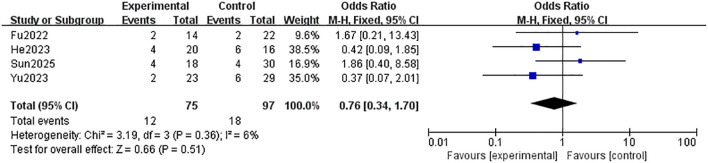
Meta-analysis of fever between DEB-BACE and cBACE.

**Figure 8:**
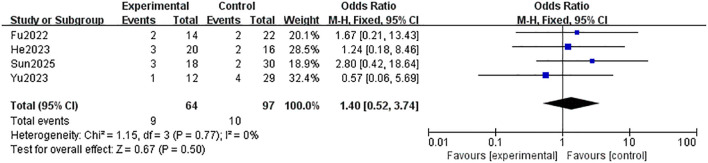
Meta-analysis of chest discomfort between DEB-BACE and cBACE.

**Figure 9:**
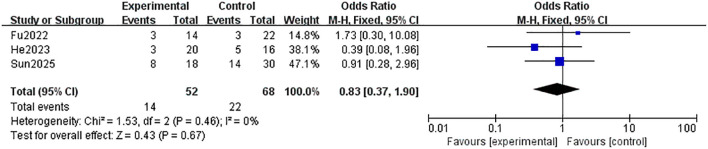
Meta-analysis of cough or sputum between DEB-BACE and cBACE.

**Figure 10:**
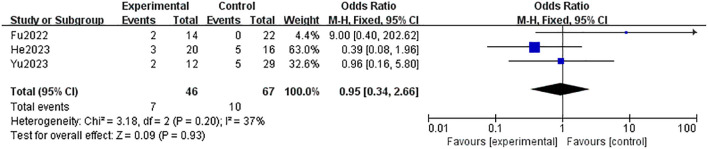
Meta-analysis of nausea or vomiting between DEB-BACE and cBACE.

Two studies evaluated hemoptysis as an outcome measure. Given the low heterogeneity observed (I^2^ = 0%), a fixed-effect model was employed for analysis. No statistically significant difference in hemoptysis incidence was found between patients receiving DEB-BACE compared to those treated with cBACE (OR = 0.31; 95% CI: 0.05–1.95; P = 0.21) (**Figure 6**). Notably, the incidence of postprocedural hemoptysis reported by Fu et al. (16.7%) was higher than that observed in the study by Yu et al. (4.9%). This discrepancy may be attributed to the inclusion of lung cancer patients with preexisting hemoptysis at baseline in the study. Four studies evaluated the incidence of fever. The results indicated no statistically significant difference between patients receiving DEB-BACE and those treated with cBACE (OR = 0.76; 95% CI: 0.34–1.70; P = 0.51). Due to low heterogeneity (I^2^ = 6%), a fixed-effect model was applied (**Figure 7**). The meta-analysis of four studies demonstrated no statistically significant difference in the incidence of chest discomfort between patients receiving DEB-BACE and those treated with cBACE (OR = 1.40 95% CI: 0.52–3.74; P = 0.50, I^2^ = 0%) (**Figure 8**). Pooled analysis of three studies (n = 120) revealed no significant difference in the incidence of cough or sputum between patients undergoing DEB-BACE (26.9%) compared to cBACE (32.4%) (OR = 0.83; 95% CI: 0.37–1.90; P = 0.67), with a fixed-effect model (I^2^ = 0%) (**Figure 9**). Three studies evaluated the incidence of postoperative nausea or vomiting, with no significant difference between groups (OR = 0.95, 95% CI: 0.34–2.66; P = 0.93; I^2^ = 37%; **Figure 10**).

## 4 Discussion

The management of lung cancer continues to present substantial clinical challenges. Disease progression and treatment-related toxicities, including myelosuppression, hemoptysis, radiation pneumonitis, and immune-mediated pneumonitis, can compromise treatment tolerance and quality of life, while also imposing substantial economic and psychological burdens on patients and their families (15). In this context, DEB-BACE represents a potentially useful locoregional treatment strategy for selected patients with lung cancer. The present analysis suggests that DEB-BACE may improve short-term tumor response without a clear increase in the assessed adverse events, although the certainty of evidence remains limited.

Transarterial interventional therapy has been widely adopted in the management of hepatocellular carcinoma and is increasingly utilized in cholangiocarcinoma (16), colorectal cancer (17), and hepatic metastases (18), etc. Lung cancer is characterized by a dual blood supply system, consisting of both the pulmonary artery and the bronchial arteries. Eldridge et al. proposed that while the pulmonary circulation may fulfill the metabolic requirements during early tumor development, subsequent growth and progression of lung cancer depend on neovascular support derived from the highly proliferative bronchial circulation (19). Researchers have indicated that in pulmonary malignant tumors, bronchial arterial flow predominates while pulmonary circulation contributes a relatively lower proportion of perfusion (20). This hemodynamic distribution provides a theoretical basis for the application of DEB-BACE in the treatment of pulmonary malignancies.

This meta-analysis compares outcomes between DEB-BACE and cBACE in lung cancer patients. The results indicate that the DEB-BACE was associated with higher pooled ORR and DCR at 1–3 months and 6 months. The efficacy of interventional therapy for tumors hinges on its capacity to sustain effective drug concentrations within the tumor, achieve prolonged drug release, and minimize damage to surrounding normal tissues (21). Drug-eluting beads can establish stable ionic bonds with chemotherapeutic agents, enabling slow and sustained drug release into the tumor via an ion-exchange mechanism. Namur et al. (22) reported that in an animal model, 57% of the chemotherapeutic agent was retained at 28 days, with 11% remaining on the drug-eluting microspheres after 3 months, continuously mediating tumor necrosis. In another study involving six patients who underwent transarterial chemoembolization with epirubicin-loaded microspheres prior to liver transplantation, local tissue concentrations of the chemotherapeutic agent were shown to remain above the cytotoxic threshold for 32–36 days post-procedure (23). However, this study is limited by the relatively small number of included studies and patient cohorts, as well as a scarcity of high-quality randomized controlled trials—issues that require urgent resolution. These limitations also explain why most clinical guidelines and consensus statements offer only limited or cursory recommendations regarding DEB-BACE.

In the analysis of adverse events, including hemoptysis, fever, chest discomfort, cough/sputum production, and nausea/vomiting, no statistically significant differences were observed between the DEB-BACE and cBACE groups. Cough or sputum production was numerically less frequent after DEB-BACE (26.9%) than after cBACE (32.4%), but this difference was not statistically significant. From a pharmacological perspective, rapid local exposure to high concentrations of free chemotherapeutic agents during conventional infusion-based treatment may theoretically contribute to mucosal irritation, inflammatory injury or infection-related vulnerability, although this mechanism was not directly tested in the included comparative studies. More generally, chemotherapy-related pulmonary complications may arise from drug toxicity, immunosuppression-related infection and immune-mediated injury (24). A systematic review by Wang et al., encompassing 11 studies and 510 NSCLC patients, demonstrated the absence of grade ≥3 adverse events associated with cBACE (25). As shown in the study by Bi et al., among patients with stage III-IV lung cancer who underwent DEB-BACE, 34.5% experienced grade 1 nausea or vomiting and 6.9% had grade 1 chest pain after the procedure, both of which were well controlled within 3–5 days. Notably, hemoptysis in all patients resolved within 1–3 days, and 12 patients showed significant improvement in cough or expectoration symptoms (26).

In addition, DEB-BACE has also been explored in combination with systemic or local therapies, but these studies are mostly single-arm or non-randomized and should not be interpreted as proof of additive benefit. Xu et al. reported that bronchial arterial interventional therapy combined with PD-1 blockade was associated with longer survival in patients with advanced non-small-cell lung cancer (NSCLC), although grade 3 adverse events were observed (27). Another study also revealed that in the combination of DEB-BACE and anlotinib, 15.7% of patients presented grade 3 AE (28). Compared with conventional chemotherapy, the combination of DEB-BACE and intercostal artery infusion chemotherapy increased the mOS of non-small cell lung cancer (NSCLC) patients with refractory malignant pleural effusion from 7 months to 13.4 months (7). In another study conducted by Bei et al., the 6-month and 12-month overall survival (OS) rates of patients who received DEB-BACE combined with targeted therapy and immunotherapy were 77.8% and 66.7%, respectively (29). A 69-year-old male patient with locally advanced NSCLC underwent DEB-BACE therapy, followed by lobectomy; postoperative pathology revealed a pathological complete response (30). One study involving 77 patients with advanced non-small cell lung cancer who were refractory to or ineligible for standard treatments demonstrated that sequential combination therapy with microwave ablation followed by DEB-BACE resulted in superior local tumor control compared to DEB-BACE alone (31).

This meta-analysis has several limitations. First, the number of included studies and patient populations is relatively small. Although the exclusion of single-arm studies and non-English literature helped reduce methodological heterogeneity and publication bias, it may have also led to the omission of some high-quality research. Second, the available literature is predominantly contributed by researchers from China. While China’s high incidence and mortality rates of lung cancer have motivated active research in this field, the geographical concentration of studies may introduce selection bias and affect the generalizability and external validity of the findings. Third, comparator definitions were heterogeneous. Some studies used bronchial arterial infusion chemotherapy followed by embolization with conventional non-drug-eluting materials, whereas Shang et al. used bronchial arterial infusion chemotherapy without reported embolization; in addition, the conventional embolic material was not clearly reported in some studies. Fourth, the present study only evaluated prognostic outcomes at short-term (1–3 months) and mid-term (6 months) follow-up. However, the long-term survival benefits of DEB-BACE compared to cBACE have not been investigated in the current literature. Finally, prior treatment history and disease recurrence status may represent notable effect modifiers in the comparative evaluation of DEB-BACE versus cBACE. In recurrent or refractory disease, where tumor heterogeneity and treatment-related clonal selection may be more pronounced (32), the sustained-release mechanism of DEB-BACE may be particularly relevant; however, this hypothesis requires prospective validation. Furthermore, patients with prior treatment exposure frequently demonstrate compromised bone marrow reserve and heightened cumulative systemic toxicity risk (33), rendering the localized drug delivery profile of DEB-BACE particularly advantageous in this subgroup. Future well-designed RCTs with stratification by prior treatment history are warranted to validate these findings.

## 5 Conclusion

This meta-analysis suggests that receiving DEB-BACE is associated with higher pooled ORR and DCR at 1–3 months and 6 months than cBACE or conventional comparator treatment in the available China-based comparative studies. The available safety data did not show a significant increase in the assessed common adverse events, but small samples and wide CIs limit inference. Because the evidence is small, predominantly observational, geographically restricted and affected by comparator heterogeneity, the findings should be interpreted cautiously. Well-designed, high-quality randomized controlled trials with standardized protocols are urgently needed to further validate the efficacy and safety of DEB-BACE and advance the field of interventional therapy for lung cancer.

## Data Availability

The original contributions presented in the study are included in the article/supplementary material. Further inquiries can be directed to the corresponding authors.
